# Intraoperative Extracorporeal Life Support for Bilateral Sequential Lung Transplantation

**DOI:** 10.3390/jcm14238315

**Published:** 2025-11-23

**Authors:** Tomislav Kopjar, Feda Dzubur, Dorian Hirsl, Goran Glodic, Goran Madzarac, Mislav Planinc, Jasna Spicek Macan, Zeljko Colak, Hrvoje Gasparovic, Miroslav Samarzija

**Affiliations:** 1 Department of Cardiac Surgery, University Hospital Center Zagreb, 10000 Zagreb, Croatia; 2 School of Medicine, University of Zagreb, 10000 Zagreb, Croatia; 3 Department of Lung Diseases, University Hospital Center Zagreb, 10000 Zagreb, Croatia; 4 Department of Thoracic Surgery, University Hospital Center Zagreb, 10000 Zagreb, Croatia; 5 Department of Anesthesiology, University Hospital Center Zagreb, 10000 Zagreb, Croatia

**Keywords:** lung transplantation, extracorporeal life support, extracorporeal membrane oxygenation, primary graft dysfunction, survival

## Abstract

**Background/Objectives**: The use of intraoperative venoarterial extracorporeal life support (VA ECLS) has traditionally been used to support unstable patients undergoing complex lung transplantation. More evidence is emerging that the use of intraoperative VA ECLS may be beneficial for all patients undergoing lung transplantation. The aim of this study was to report the safety and feasibility of lung transplantation with the routine use of central VA ECLS. **Methods**: In this single-center retrospective observational study, all consecutive patients undergoing lung transplantation from April 2021 until September 2025 were included. Early outcomes and the incidence of primary graft dysfunction were evaluated with the International Society for Heart and Lung Transplantation criteria at 72 h after transplantation. Survival and chronic lung allograft dysfunction (CLAD)-free survival were reported with Kaplan–Meier estimates and 95% confidence intervals (CIs). **Results**: During the study period, 35 patients were successfully transplanted with the aid of central VA ECLS. There were no complications associated with intraoperative ECLS. One revision surgery was performed for immediate postoperative bleeding, and one for bronchial anastomosis air leak. Operative mortality occurred in three patients (8.6%). The median in-hospital stay was 30 (25–43) days. Severe primary graft dysfunction at 72 h was observed in four (11.4%) patients. Survival and CLAD-free survival at 1-, 3-, and 5-years following surgery were 85% (95% CI [74–98]), 74% (95% CI [59–92]), 67% (95% CI [28–82]), and 82% (95% CI [70–96]), 52% (95% CI [37–74]), 36% (95% CI [11–59]), respectively. **Conclusions**: Lung transplantation can safely be performed with the aid of central VA ECLS, with a low rate of primary graft dysfunction and favorable long-term outcomes. Further follow-up studies and greater experience are needed to make inferences on the long-term outcomes. This technique is relatively recent and evolving, representing an innovative intersection of advanced supportive technology with transplant surgery, potentially broadening indications and improving success rates.

## 1. Introduction

Lung transplantation has long been regarded as a life-saving procedure for patients with end-stage lung diseases, offering a chance for extended survival and improved quality of life; however, it is also associated with significant postoperative morbidity and mortality, with survival rates inferior to those of other solid organ transplants. This early mortality and morbidity are primarily attributed to primary graft dysfunction (PGD), a multifactorial process influenced by both donor- and recipient-related risk factors [1]. Despite favorable results in recent years, lung transplantation continues to be limited by poor long-term outcomes, which are related to chronic lung allograft dysfunction (CLAD) [2].

Ensuring optimal intraoperative handling of donor lungs is crucial for preserving the functional integrity of the lung graft. Lack of a standardized surgical approach contributes to disparities in results, particularly regarding the need for intraoperative extracorporeal life support (ECLS). In recent years, the integration of ECLS during lung transplantation has gained prominence as a transformative advancement in the management of these critically ill patients [3].

Sequential double-lung transplantation enables an off-pump implantation without ECLS. Historically, a perfusion-guided implantation sequence has been recommended for off-pump double-lung transplantation [4]. Venoarterial (VA) configuration of ECLS provides continuous respiratory and circulatory support during lung transplantation. This is particularly beneficial for those transplant candidates with concomitant cardiac issues or pulmonary hypertension, where traditional ventilatory support and pharmacological interventions prove insufficient [5]. Whether the application of this technology benefits all transplant candidates remains to be elucidated.

Despite promising results, the intraoperative application of ECLS remains a subject of ongoing debate. We hypothesize that the widespread utilization of central VA ECLST during lung transplantation will be advantageous for our patients, as evidenced by a low incidence of PGD at 72 h post-surgery. While it guarantees intraoperative lung protective ventilation, hemodynamic stability, and controlled graft reperfusion—minimizing patient and graft stress—it also carries risks associated with cannulation, heparinization, and inflammatory response.

With this article, we aimed to explore the results of bilateral sequential lung transplantation performed with the aid of intraoperative ECLS support. We examined the clinical outcomes, benefits, and challenges associated with its use and the effect on operative mortality, PGD, and long-term survival.

## 2. Materials and Methods

### 2.1. Study Population

This single-center retrospective observational study included all consecutive patients undergoing lung transplantation at the University Hospital Center Zagreb in Zagreb, Croatia, since the beginning of the program in April 2021 until September 2025. Patients were followed up monthly in the outpatient clinic until September 2025, when follow-up was terminated for this study. Notably, no patients were lost to follow-up. The only inclusion criterion was surgery for lung transplantation, and there were no exclusion criteria. Both adult and pediatric patients were included in this study, although there was only one pediatric case. The institutional review board of the University Hospital Center Zagreb approved this study, and it was conducted in accordance with the Declaration of Helsinki. Since this study was retrospective, written informed consent was waived. Individual medical records were reviewed for demographic, clinical, and laboratory data. Patient demographic and operative characteristics, as well as outcomes during the follow-up period—which was completed in September 2025—were reported.

### 2.2. Organ Procurement

Donor-lung organ procurement for lung transplantation was conducted in a standardized manner. Before aortic cross-clamping, all donors received prostaglandin E1 through their central venous lines. After cross-clamping, grafts were flushed antegradely with 6 L of Perfadex Plus solution (XVIVO Perfusion, Molndal, Sweden). The Perfadex Plus solution was supplemented with another dose of prostaglandin E1 before flushing. Once the lungs were explanted, a final visual inspection was conducted. If the lungs were deemed adequate, at this moment, the recipient was taken to the transplant center’s operating room. Following lung explantation in the procurement center, the lungs were placed on the back table and flushed once again retrogradely through the left atrium with three liters of Perfadex Plus solution. This retrograde flush aims to remove any debris or thrombi from the pulmonary artery bed. After retrograde flushing, the lungs were split, individually wrapped, and stored in portable coolers set for cold static preservation on ice during transportation.

### 2.3. Lung Transplantation

The recipients arrived at the operating room once the final donor organ approval was granted. A standardized strategy for bilateral sequential lung transplantation on ECLS was implemented. After making a clamshell incision, the pericardium was opened, and patients received a single bolus dose of 50–60 units/kg heparin, based on surgeons’ preference. Anticoagulation was not routinely monitored, and heparin was not readministered during surgery. An EOPA cannula (Medtronic Inc., Minneapolis, MN, USA) and a curved-tip wired venous drainage cannula were inserted into the ascending aorta and right atrium, respectively. These cannulas were connected to a Cardiohelp (Getinge AB, Goteborg, Sweden) or Biomedicus (Medtronic Inc., Minneapolis, MN, USA) centrifugal pump. Hollow-fiber membrane oxygenators, also from Getinge, were incorporated into the heart–lung support or the permanent life support set. Biocompatible ECLS surface coating lines were used. The flow rate was set to 50% of the recipient’s cardiac output, ventilation was reduced to 6 mL/kg tidal volume (lung protective ventilation), and a bilateral sequential lung transplantation was performed. During the de-airing of the newly implanted lung, the ECLS flow was temporarily reduced to 1 L/min. During second-side implantation, ECLS flow was adjusted to maintain pulmonary systolic pressures of 25 mmHg; however, it was always ensured that the flow was pulsatile and within the normal range of end-tidal carbon dioxide (CO_2_). After both sides were implanted, the ECLS flow was gradually reduced, the cannulas were clamped, and the arterial and venous lines were cut and reconnected. The tubing was kept sterile, recirculating on the operating room table. This allowed for possible reinstitution of the ECLS if needed. Following predefined criteria for extended ECLS, the function of the implanted graft was evaluated before and after closing the chest, as described by Hoetzenecker et al. [5]. In case an extended ECLS support was required, it was initiated via the femoral vessels in a VA configuration until recovery. The prolonged support usually lasted over the next 72 h. Intraoperatively, all patients were administered methylprednisolone, and the induction therapy utilized alemtuzumab (Campath, Sanofi, MA, USA) upon arrival at the intensive care unit (ICU). The immunosuppression protocol for our lung transplant patients was described previously and remained unchanged throughout the study duration [6].

### 2.4. Outcome Measures

The primary outcome of interest was operative mortality, defined as death within 30 days after surgery or in-hospital death. Secondary outcomes of interest included in-hospital stay, ICU stay, duration of mechanical ventilation, rates of PGD at 0, 24, 48, and 72 h following surgery, long-term survival, and CLAD-free survival. To assess PGD, a grading system was developed, incorporating arterial oxygen partial pressure (PaO_2_) to fractional inspired oxygen (FiO_2_) ratio and chest radiograph findings. This system allowed each patient to be graded with a PGD grade of 0–3, as outlined in the 2017 consensus group statement of the International Society for Heart and Lung Transplantation (ISHLT) [7]. Presence of PGD in the first 72 h after transplantation was used to dichotomize the cohort into two groups. Chest radiographs were independently rated by trained radiologists. The radiologists were not involved in the clinical management of the patients and were blinded to their momentary clinical condition. If bilateral infiltrations were absent, a PGD of 0 was documented. In contrast, radiologic signs of reperfusion edema led to the use of P/F ratios to differentiate between PGD 1 and 3: PGD1: P/F ratio > 300 mmHg; PGD2: P/F ratio 200–300 mmHg; and PGD3: P/F ratio < 200 mmHg. Patients on extended postoperative ECLS were classified as PGD3 if chest radiographs demonstrated bilateral infiltrations. Chronic lung allograft dysfunction is a broad term used to describe a significant decline in lung function after lung transplantation, in the absence of other identifiable causes. In our study, we defined CLAD as a substantial and persistent decline of at least 20% in forced expiratory volume in the first second (FEV1) compared to the post-transplant baseline [8]. The baseline FEV1 is calculated as the average of the two maximal post-transplant FEV1 values that are at least three weeks apart.

### 2.5. Statistics

Continuous variables were expressed as mean ± standard deviation (SD) or median with interquartile range (IQR), while categorical variables were described with frequencies and proportions. To assess the distribution of continuous variables, the Shapiro–Wilk test for normality was applied to determine which variables were normally distributed and which were non-normally distributed. Normally distributed variables were presented with mean ± SD, and non-normally distributed as median with IQR. The Mann–Whitney U test was used for non-parametric comparisons, and the independent Student’s *t*-test without equal variance assumption was used for parametric group comparisons. For categorical data, Fisher’s exact test was used according to the expected sample sizes in the contingency table. Two-sided *p* values of less than 0.05 were considered statistically significant.

Overall survival was defined as the time from lung transplantation to death from any cause. Patients were censored at the last date known to be alive. There were no patients lost during follow-up. Alive or dead status was known for each patient at the end of the follow-up. Chronic lung allograft dysfunction-free survival was defined as the time from lung transplantation to the onset of CLAD or death from any cause. In the CLAD-free survival analysis, patients were censored at the last date known to be alive and free of CLAD. All the survival curves with their respective 95% confidence intervals (CIs) were plotted using the Kaplan–Meier method. These survival curves were used to calculate the 1- and 3-year survival estimates.

We used parametric survival modeling to calculate the 5-year survival and CLAD-free survival probabilities. The SurvInt tool was used for survival modeling [9]. This tool creates several parametric survival models fitted to two interpolation points from the individual patient data. The Kaplan–Meier estimates at 12 and 36 months were used as the interpolation points from our dataset. The best-fit parametric model was chosen, and the 5-year estimates were extrapolated from it. The probabilistic sensitivity analysis was used to assess the impact of uncertainty in 5-year survival estimates, and the corresponding 95% CIs were calculated after 1000 iterations.

The statistical analyses were performed with IBM SPSS Statistics version 31 (IBM Corp., Armonk, NY, USA) and R version 4.5.0 (R Foundation for Statistical Computing, Vienna, Austria).

## 3. Results

### 3.1. Baseline Characteristics of the Cohort

A total of 35 patients underwent transplant during the study period. The median age of the cohort was 55 (50–59) years. Chronic obstructive pulmonary disease was the most common indication for lung transplantation, followed by idiopathic pulmonary fibrosis and pulmonary hypertension. The median lung allocation score was 37 (33–44). In total, two (5.7%) were high-urgency cases on mechanical ventilation for which exceptional lung-allocation scores were granted. None of the patients required bridging with ECLS at the time of transplantation. Detailed patient demographics and characteristics are provided in Table 1.

### 3.2. Donor Characteristics

Mean donor age was 41 ± 13 years. All organs were retrieved from donors who were brain dead, with intracerebral bleeding being the most common cause of death. In total, nine (25.7%) donors had a smoking history, five (14.3%) had an abnormal radiograph, and two (5.7%) had reported aspiration. In eight (22.9%), an abnormal bronchoscopy with distal secretions was documented. Donors were on mechanical ventilation for a median time of 3.7 (2.4–5.9) days before procurement. Mean value of arterial pO_2_ was 456 ± 100 mmHg, and the median for arterial pCO_2_ was 40 (34–44) mmHg. Remaining detailed donor characteristics are presented in Table 2.

### 3.3. Early Outcomes and Primary Graft Dysfunction

Mean graft ischemic time was 416 ± 80 min—360 ± 55 min for the first and 469 ± 63 min for the second lung graft. Female-to-male gender mismatch was observed in 13 (37.1%) cases. Donor-positive and recipient-negative cytomegalovirus mismatch was observed in two (5.7%) cases, and non-identical but compatible ABO matching was present in six (17.1%) cases. There were no ABO-incompatible transplants.

All cases were performed on central VA ECLS. There were no complications associated with intraoperative central ECLS. Lung resection was required in 10 (28.6%) cases, consisting of atypical resection of the middle lobe on the right side and the lingula on the left side. One revision surgery was performed for immediate postoperative bleeding, and one for bronchial anastomosis air leak; both patients had an uneventful postoperative course after the revision. None of the patients had a wound (clamshell) infection or dehiscence. Three patients died during the initial hospital stay, accounting for 8.6% operative mortality. Remaining early outcomes are reported in Table 3.

Prolonged postoperative ECLS was necessary in four patients (11.4%); this was related to one case of lower-limb ischemia. The patients required embolectomy of the right common femoral and external iliac artery after weaning from ECLS. Timely embolectomy was performed, and there were no long-term sequelae to the limb. There were no other observed complications associated with prolonged postoperative ECLS.

**Figure 1:**
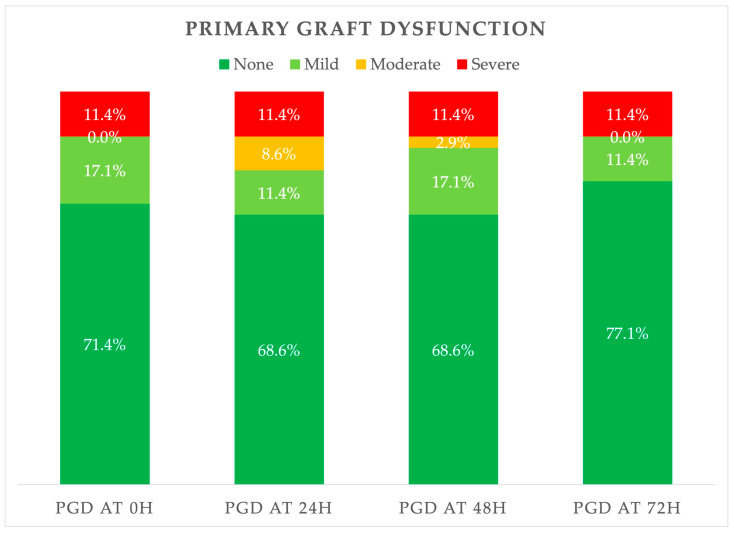
Primary graft dysfunction.

Rates of PGD are depicted in Figure 1. At 72 h after surgery, 31 (88.6%) patients had no or mild PGD, and 4 (11.4%) had severe PGD. There were no patients with moderate PGD at 72 h after surgery, and there were 16 (45.7%) patients that were extubated at that time point, requiring only oxygen insufflation via a nasal catheter.

We compared all the previously reported recipient and donor characteristics, as well as the early outcomes between patients that developed PGD and those that did not develop it. Patients with PGD had more lung resections [3 (13.6%) vs. 7 (53.8%), *p* = 0.02], longer second-lung-graft ischemia (451 ± 55 vs. 504 ± 63, *p* = 0.021), and higher operative mortality [0 (0%) vs. 3 (32.1%), *p* = 0.044]. Primary graft dysfunction was also associated with death during follow-up [2 (9.1%) vs. 6 (46.2%), *p* = 0.032]. Remaining comparisons that were significant are outlined in Table 4.

### 3.4. Long-Term Survival

**Figure 2:**
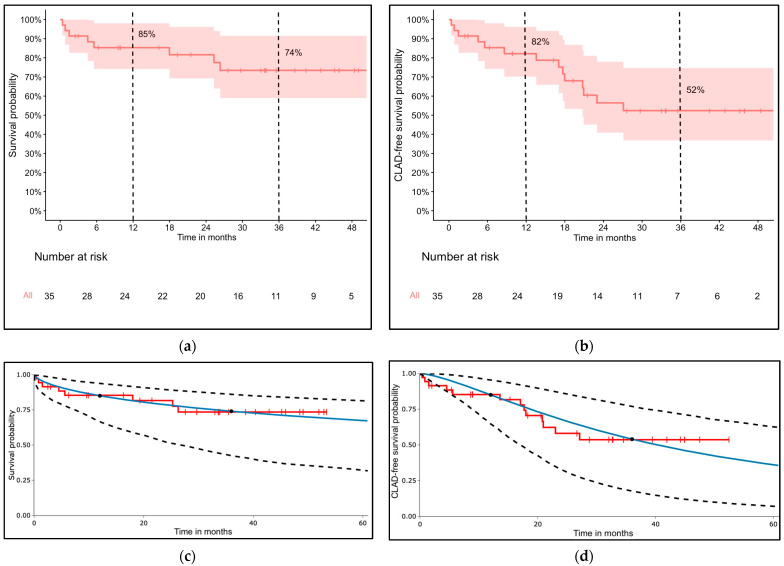
Survival curves and 95% confidence intervals for (**a**) overall survival; (**b**) chronic lung allograft dysfunction-free survival; (**c**) 5-year extrapolated survival probability from the log-logistic model marked with blue line; (**d**) 5-year extrapolated chronic lung allograft dysfunction-free survival probability from the log-logistic model marked with blue line.

One-year and three-year survival rates calculated by the Kaplan–Meier method were 85% (95% CI [74–98]) and 74% (95% CI [59–92]), respectively (Figure 2a). Chronic lung allograft dysfunction-free survival rates at one and three years were 82% (95% CI [70–96]) and 52% (95% CI [37–74]), respectively (Figure 2b).

After a visual inspection of the survival models based on the interpolation points at 12 and 36 months, the log-logistic model fit our data best (Figure 2c,d). The five-year survival and CLAD-free survival estimates based on the log-logistic model were 67% (95% CI [32–82]) and 36% (95% CI [11–59]), respectively.

## 4. Discussion

End-stage lung disease unresponsive to medical management remains a significant challenge in respiratory medicine. Lung transplantation has emerged as a crucial surgical intervention, providing the potential for symptom relief and improved survival. When performed in patients with severe respiratory compromise, ECLS often plays a pivotal role in hemodynamic stability and adequate oxygenation during transplantation.

Lung transplantation is the gold standard therapy for end-stage lung diseases that are refractory to more conservative therapies. However, survival after lung transplantation is worse than survival after other solid organ transplantations. The survival of lung-transplanted patients has been impaired by the development of PGD, acute infections, malignancies, acute lung allograft dysfunction, and CLAD. At the beginning of our program in 2021, we opted for the strategy of lung transplantation on central VA ECLS based on the successes published previously with this approach in the most challenging cases [5]. The results of lung transplantation in our center are encouraging. We report low operative mortality and promising long-term survival. Long-term survival was based on modeled data. International registry data report a median survival time of 7.8 years for bilateral lung transplantation in adult patients [10]. Likewise, 1- and 5-year survival rates are reported at 85 and 59%, respectively [11]. These survival rates are comparable to our data. The 1-year survival estimate in our patient cohort was 85%. Based on 1- and 3-year observed survival rates of our patient cohort, the 5-year estimate of survival probability was extrapolated from the log-logistic survival model and estimated at 67%. Single-center data reported from a high-volume center reveal a significant improvement in the 5-year unadjusted survival rate over recent years; specifically, survival increased from 54% between 1993 and 1997 to 72% between 2010 and 2019 [12].

Chronic lung allograft dysfunction is the leading cause of mortality beyond the first year following lung transplantation. It accounts for up to 30% of deaths during follow-up, and remains the primary concern in long-term outcomes [2]. Not only does it impact survival, severe CLAD also impairs health-related quality of life [13]. While 1-year CLAD-free survival rates in our cohort are promising, the true effect of CLAD is observed in the long-term.

One of the primary advantages of combining lung transplantation with ECLS is its potential to serve as a life-saving procedure for carefully selected patients. It provides critical support that enables complex surgical interventions to be conducted safely, even in the setting of severe hypoxia or hemodynamic instability. In many lung transplantation centers, ECLS is replacing cardiopulmonary bypass as the primary choice for intraoperative support [14]. Centers performing lung transplantation in stable patients without support report about 50% necessity of ECLS during surgery; this may vary based on patient selection and institutional protocols. Furthermore, ECLS can act as a bridge to transplantation, affording patients additional time for preoperative optimization and assessment [15]. Success in these contexts can dramatically restore lung function, alleviate symptoms such as hypoxia and dyspnea, and substantially enhance quality of life, positioning this approach as a vital recourse for end-stage lung diseases.

Preemptive intraoperative VA ECLS has been associated with superior outcomes compared to transplantation without any support [5]. In challenging cases, such as those involving pulmonary hypertension and patients with questionable graft function at the end of implantation, prolonged postoperative VA ECLS provides an extended period for graft improvement. Lung transplantation with intraoperative VA ECLS support provides enhanced reperfusion conditions that might translate into superior primary graft function compared to off-pump techniques [16]. Our cohort was predominantly composed of young patients with chronic obstructive pulmonary disease, which could influence the likelihood of requiring support and may overlap with cohorts managed without routine use of ECLS. Future studies that directly compare these populations, considering clinical characteristics, would clarify the impact of VA ECLS on outcomes.

Primary graft dysfunction is a clinical syndrome occurring within the first 72 h after lung transplantation and is characterized clinically by progressive hypoxemia and patchy alveolar infiltrates on chest radiographs. It remains one of the main risk factors associated with poor early outcomes after lung transplantation. Severe PGD is still reported in up to 20% of patients within the first 72 h following lung transplantation [7]. In our series, severe PGD at 72 h following transplant was 11%. Unsurprisingly, severe PGD has been associated with increased operative mortality and impaired 1-year survival rates [1]. Baseline and donor data comparing patients with and without PGD were mostly comparable in our cohort. The 6 min walking distance was shorter in the PGD group, potentially confounding the results of the comparison. We report increased operative mortality and mortality during follow-up among patients with PGD during the initial 72 h following transplantation. This is in accordance with previous reports claiming an association of PGD with early morbidity and mortality after lung transplantation. However, we did not find an association between PGD and CLAD. The association of PGD and CLAD remains a matter of ongoing debate—some papers support this association while others do not [17].

Several risk factors contribute to PGD in patients following lung transplantation; these are associated with donor or recipient characteristics, as well as technical details of procurement and transplantation. Prolonged lung graft ischemia has been associated with PGD in previous studies [18]. In our study, overall lung graft ischemic times were similar in PGD and non-PGD patients, albeit with a borderline *p* value of 0.057. We could say that a trend towards longer graft ischemia was observed among PGD patients. Looking at each individual lung, second-lung ischemic times were longer among those developing PGD within 72 h after transplantation.

Donor-to-recipient gender mismatch has been associated with worse outcomes in solid organ transplantations. In our series, female donor to male recipient was reported in 37% of cases. Whether gender mismatch in lung transplantation is associated with worse outcomes is unclear since the data are relatively scarce [19]. In the ISHLT report, donor–recipient gender mismatch was a risk factor for 1-year mortality, and female recipient gender was a risk factor for 5-year mortality [20]. However, in the more recent 20th ISHLT official report, donor or recipient gender was not identified as a risk factor [21].

Lung transplantation on ECLS is not without its drawbacks. The procedure’s high morbidity and mortality risks are well-documented, with complications such as bleeding, infection, and thrombotic events posing significant challenges. Prolonged postoperative ECLS itself may contribute to additional complications, such as limb ischemia, bleeding, or neurological injury, which can adversely influence outcomes. Intraoperative central VA ECLS did not lead to any overt complications in our series, although prolonged postoperative ECLS did lead to one case of lower-limb ischemia. The invasiveness of the lung transplant surgery, coupled with the complexity of maintaining ECLS support, necessitates a highly specialized, multidisciplinary team and considerable healthcare resources. These factors underscore the procedure’s resource-intensive nature and limit its widespread accessibility.

Post-transplant patients face ongoing risks, including chronic rejection and infections related to immunosuppression. The persistent shortage of donor lungs limits the availability of lung transplantation as a definitive therapy, often resulting in prolonged wait times and deterioration in the patient’s condition during this period. To address this issue, many centers are considering marginal donor lungs. Studies imply comparable post-transplant outcomes between ex vivo donor-lung perfusion and standard donor-lung procurement, although the ex vivo lung perfusion was employed on marginal donor lungs of poorer quality [22,23]. This strategy could potentially improve donor-lung use and increase the success rate of transplantation. Strategies for maximizing lung utilization in donors after brain and cardiac death are vital to addressing the global shortage of suitable lungs for transplantation [24].

This retrospective observational study lacks a dedicated control group, which introduces inherent limitations. It is also confounded by the retrospective observational study design. Additionally, the results of this study should be interpreted with caution due to the rather small patient sample. The small sample size precludes the calculation of regression statistics, and no power analysis was performed to determine the minimal sample size for this study. However, this study does have several strengths. It established a dedicated follow-up outpatient clinic with no patients lost to follow-up, ensuring comprehensive data collection. Furthermore, the prospective nature of the data collection enhances its granularity. Future research should explore the association between each individual lung of PGD patients and the graft ischemic times.

In conclusion, lung transplantation supported by ECLS remains a vital yet complex therapeutic option for patients with severe lung failure. Its success hinges on careful patient selection, meticulous perioperative management, and a multidisciplinary approach that maximizes benefits while minimizing risks. As technology and immunosuppression continue to advance, refining this approach holds promise for improving long-term outcomes in this vulnerable patient population. We believe that intraoperative VA ECLS support might enhance the overall results of lung transplantation.

## Figures and Tables

**Table 1:** Patient demographics and characteristics.

	All Subjects (*n* = 35)
Age (years)	55 (50–59)
<40	3 (8.6%)
40–60	26 (74.3%)
>60	6 (17.1%)
Female	7 (20%)
Height (cm)	1.72 ± 0.1
Body mass index (kg/m^2^)	23.8 ± 3
<18.5	1 (2.9%)
18.5–24.9	20 (57.1%)
25.0–29.9	13 (37.1%)
30.0–40	1 (2.9%)
Arterial hypertension	7 (20%)
Pulmonary hypertension	15 (42.9%)
Diabetes	4 (11.4%)
Dyslipidemia	2 (5.7%)
Long-term oxygen therapy (L/min)	2 (1–3.5)
Non-invasive positive pressure ventilation	6 (17)
6 min walk (m)	194 ± 107
<150	11 (31.4%)
150–300	18 (51.4%)
>300	6 (17.1%)
Underlying disease	
Chronic obstructive pulmonary disease	16 (45.7%)
Idiopathic pulmonary fibrosis	13 (37.1%)
Pulmonary hypertension	2 (5.7%)
Cystic fibrosis	1 (2.9%)
Others	3 (8.6%)

Categorical data are expressed as number with (%), and continuous variables as mean ± standard deviation or median with interquartile range.

**Table 2:** Donor characteristics.

	All Subjects (*n* = 35)
Age (years)	41 ± 13
<40	13 (37.1%)
40–60	19 (54.3%)
>60	3 (8.6%)
Female	17 (49%)
Height (cm)	1.73 ± 0.09
Body mass index (kg/m^2^)	25.3 ± 3.5
<18.5	1 (2.9%)
18.5–24.9	15 (42.9%)
25.0–29.9	17 (48.6%)
30.0–40	3 (8.6%)
Arterial hypertension	9 (25.7%)
Diabetes	3 (8.6%)
Intravenous drug abuse	1 (2.9%)
Alcohol abuse	9 (25.7%)
Cytomegalovirus	31 (88.6%)

Categorical data are expressed as number with (%), and continuous variables as mean ± standard deviation or median with interquartile range.

**Table 3:** Early outcomes.

	All Subjects (*n* = 35)
Length of mechanical ventilation (days)	3.5 (2.5–5.6)
Length of ICU stay (days)	6 (4–8)
Length of hospital stay (days)	30 (25–43)
30-day mortality	2 (5.7%)

Categorical data are expressed as number with (%), and continuous variables as mean ± standard deviation or median with interquartile range. ICU = intensive care unit.

**Table 4:** Comparison of patients with and without primary graft dysfunction.

	No PGD (*n* = 22)	PGD (*n* = 13)	*p* Value
Recipient characteristics			
Age (years)	56 (51–58)	52 (44–62)	0.933
Female	4 (18.2%)	3 (23.1%)	1.000
Height (cm)	1.73 ± 0.11	1.71 ± 0.07	0.584
Body mass index (kg/m^2^)	23.5 ± 2.9	24.5 ± 3.1	0.363
Arterial hypertension	5 (22.7%)	2 (15.4%)	0.689
Pulmonary hypertension	9 (40.9%)	6 (46.2%)	1.000
Diabetes	3 (13.6%)	1 (7.7%)	1.000
Dyslipidemia	0 (0%)	2 (15.4%)	0.131
Long-term oxygen (L/min)	2 (1–2)	2 (2–4)	0.091
Non-invasive positive pressure ventilation	2 (9.1%)	4 (30.8%)	0.166
6 min walk (m)	234 ± 100	126 ± 82	0.002
Chronic obstructive pulmonary disease	12 (54.5%)	4 (40.8%)	0.293
Idiopathic pulmonary fibrosis	8 (36.4%)	5 (38.5%)	1.000
Pulmonary hypertension	0 (0%)	2 (15.4%)	0.131
Cystic fibrosis	0 (0%)	1 (7.7%)	0.371
Other	2 (9.1%)	1 (7.7%)	1.000
Donor characteristics			
Age (years)	39 ± 14	45 ± 11	0.198
Female	9 (40.9%)	8 (61.5%)	0.305
Height (cm)	1.74 ± 0.1	1.72 ± 0.05	0.425
Body mass index (kg/m^2^)	25.8 ± 4.1	24.6 ± 2.2	0.288
Arterial hypertension	7 (31.8%)	2 (15.4%)	0.431
Diabetes	2 (9.1%)	1 (7.7%)	1.000
Intravenous drug abuse	1 (4.5%)	0 (0%)	1.000
Alcohol abuse	5 (22.7%)	4 (30.8%)	0.698
Cytomegalovirus	19 (86.4%)	12 (92.3%)	1.000
Outcomes			
Length of mechanical ventilation (days)	2.7 (2.1–3.6)	5.8 (5.2–9.6)	<0.001
Length of ICU stay (days)	5 (4–6)	8 (6–10)	0.004
Length of hospital stay (days)	27 (24–35)	41 (29–61)	0.057
30-day mortality	0 (0%)	2 (15.4%)	0.131

Categorical data are expressed as number with (%), and continuous variables as mean ± standard deviation or median with interquartile range. ICU = intensive care unit.

## Data Availability

The original contributions presented in this study are included in the article Further inquiries can be directed to the corresponding author.

## Abbreviations

The following abbreviations are used in this manuscript:

CI	Confidence interval
CLAD	Chronic lung allograft dysfunction
ECLS	Extracorporeal life support
ICU	Intensive care unit
ISHLT	International Society for Heart and Lung Transplantation
PGD	Primary graft dysfunction
VA	Venoarterial
